# Predicting the response of patients with advanced urothelial cancer to methotrexate, vinblastine, Adriamycin, and cisplatin (MVAC) after the failure of gemcitabine and platinum (GP)

**DOI:** 10.1186/s12885-015-1825-5

**Published:** 2015-10-27

**Authors:** Ki Hong Kim, Sung Joon Hong, Kyung Seok Han

**Affiliations:** Department of Urology and Urological Science Institute, Yonsei University College of Medicine, 50-1, Yonsei-ro, Seodaemun-gu, Seoul 120-752 South Korea

**Keywords:** Urothelial carcinoma, Chemotherapy, Cisplatin, Second-line

## Abstract

**Background:**

Platinum-based systemic chemotherapy is the treatment of choice for patients with advanced urothelial carcinoma (UC). Although no chemotherapeutic regimen is established as a second-line therapy, recent studies reported that methotrexate, vinblastine, Adriamycin and cisplatin (MVAC) elicited a significant response in patients who failed gemcitabine and platinum (GP) chemotherapy. We investigated the clinical factors useful for predicting a favourable response to MVAC in UC patients who failed GP.

**Methods:**

Forty-five patients with advanced UC who received second-line MVAC chemotherapy after failure with first-line GP chemotherapy were enrolled in this study. Univariate and multivariate analyses based on Cox’s regression were performed to identify independent prognostic factors for progression-free survival (PFS) after second-line MVAC chemotherapy.

**Results:**

The median follow-up period after the first MVAC administration was 10.0 months. The median PFS and overall survival (OS) were 6.5 months (95 % confidence interval [CI]: 5.1–7.9) and 14.5 months (95 % CI, 7.4–21.4), respectively. The overall response rate was 57.8 %. The response to first-line GP chemotherapy (hazard ratio [HR], 2.500; *p* = 0.012) and patient age (HR, 1.047; *p* = 0.033) were predictors of PFS after MVAC chemotherapy.

**Conclusions:**

The response to first-line GP chemotherapy and age were independent predictors of PFS in patients who received second-line MVAC chemotherapy. This report is the first to describe independent predictors of PFS after MVAC chemotherapy.

## Background

Systemic chemotherapy is the treatment of choice for metastatic, recurrent or inoperable urothelial carcinoma (UC). Methotrexate, vinblastine, Adriamycin and cisplatin (MVAC) chemotherapy was used worldwide as the standard treatment since the first report of its efficacy in 1985 [1]. However, since gemcitabine and cisplatin (GC) chemotherapy showed similar efficacy as MVAC but with less toxicity in a large, randomized, multinational and multicentre phase III study of GC combination chemotherapy, [2] GC regimens are now used mostly as the initial systemic chemotherapy for UC.

GC chemotherapy has an excellent response rate in patients with advanced UC, and up to 20 % of individuals have achieved a complete response of long-term, disease-free survival [3]. However, most patients eventually experience disease progression or relapse after GC. Several regimens have been investigated in the second-line setting after the failure of GC, including taxanes, vinflunine, ifosfamide, ixabepilone and pemetrexed; [4–9] however, no regimen has yet achieved a competent survival benefit in the second-line setting.

Recently, a small number of clinical trials proposed that platinum-based regimens are effective in a significant portion of patients in whom platinum-based chemotherapy initially failed when the regimen was based on cisplatin but using different agents in the second-line setting. The efficacy and safety of MVAC chemotherapy as a second-line treatment after the failure of gemcitabine and platinum (GP) chemotherapy has been reported in several small-scale studies [10–13]. However, there is a lack of predictive factors available for the personalized selection of chemotherapeutic regimens in patients with UC. Therefore, we investigated the factors predictive of a favourable response to second-line MVAC chemotherapy after GP in patients with advanced UC to facilitate the development of a tailored second-line treatment strategy. Here, we investigated the predictive value of the response to first-line GP chemotherapy for selecting suitable candidates for the MVAC regimen as the second-line chemotherapy in patients with advanced UC.

## Methods

### Study population

The medical ethics committee of Severance Hospital, Yonsei University Health Care System (Seoul, Korea) approved this retrospective study. Our medical ethics committee allows exempt of informed consent if the research uses the collection or study of existing data, documents and records and these sources are publicly available and the information is recorded by the investigator in such a manner that subjects cannot be identified. From these regulations, our study was classified as exempt from the informed consent requirement because this study was based on the collection of existing publicly available data, documents and records, and we recorded all data in a manner that subjects cannot be identified. Between July 2004 and August 2014, 64 consecutive patients who received MVAC chemotherapy as the second-line treatment due to the relapse or disease progression of UC after first-line GP chemotherapy in the only metastatic setting were included in this study. Nineteen of the 64 patients were excluded because of incomplete medical records (seven patients), another synchronous metastatic malignancy (five patients), an atypical carcinoma (four patients) or a history of neoadjuvant chemotherapy (three patients). Forty-five patients were included in the final analysis.

### Treatment

Before starting chemotherapy, all patients were required to have an Eastern Cooperative Oncology Group (ECOG) performance status (PS) ≤ 2, adequate hematologic parameters (absolute granulocyte count ≥ 1,500/dL, haemoglobin ≥ 8.5 g/dL and a platelet count ≥ 100,000/dL) and sufficient hepatic (serum bilirubin ≤ 1.5 mg/dL) and renal (estimated glomerular filtration rate ≥ 50 mL/min) function. Methotrexate was given at a dose of 30 mg/m^2^ on days 1, 15 and 22, vinblastin was given at a dose of 3 mg/m^2^ on days 2, 15 and 22, Adriamycin was given at a dose of 30 mg/m^2^ on day 2 and cisplatin was given at a dose of 70 mg/m^2^ on day 2. The cycles were repeated every 28 days, and treatment continued until the disease regressed or the toxicity was intolerable.

### Response and toxicity assessment and dose modification

Basically, after the completion of three cycles, imaging tools (computed tomography scans, radionuclide bone scans or positron emission tomography) were used to evaluate the treatment response after three cycles, except for cases with symptomatic progression. Performance status, haematological parameters and liver and kidney function were measured weekly in each patient to assess for potential toxicity. The response was evaluated according to the Response Evaluation Criteria for Solid Tumours, [14] and toxicity was determined using the National Cancer Institute Common Toxicity Criteria (ver. 4.0). The platinum dose was reduced by 20–30 % in patients who experienced grade 4 haematological toxicity or grade 3/4 non-haematological toxicity.

### Outcomes

The end point of the study was PFS in patients who received MVAC chemotherapy. The PFS of patients who received MVAC chemotherapy was defined as the time from the date of the beginning of the first MVAC cycle to the date that progression was identified, death, or loss to follow-up. Progression was defined as a ≥ 20 % increase in the overall sum of the diameter of the target lesions on radiological assessments.

### Clinical data and statistical analysis

All included patients were divided into good or poor response groups based on the response to the first-line GP chemotherapy. The good response group included the complete response (CR) and partial response (PR) groups to first-line GP chemotherapy, while the poor response group included the stable disease (SD) and progression disease (PD) groups [15]. The baseline characteristics of the two groups were then compared using Chi-squared tests. PFS after MVAC chemotherapy was calculated using the Kaplan-Meier method, and statistical significance was determined using log rank tests. Age, haematological parameters, liver and kidney function, serum albumin concentration at the beginning of the first MVAC and time to progression (TTP) after first-line GP chemotherapy were included in the analysis as continuous variables. Clinical nodal status, distant metastatic status, ECOG PS at the beginning of the first MVAC and response to previous GP chemotherapy were analysed as categorical variables. Statistical analyses to identify independent predictors of progression after MVAC chemotherapy were performed using univariate and multivariate Cox’s proportional hazard regression analyses. Variables that were significant in the univariate analysis (*p* < 0.05) were entered into the multivariate model. All statistical analyses were performed using SPSS Statistics version 20.0.0 (IBM Corp., Armonk, NY, USA). For all analyses, a two-sided *p*-value <0.05 was considered to indicate statistical significance.

## Results

### Patients

The median follow-up period after the first MVAC administration was 10.0 months. The median PFS and overall survival (OS) were 6.5 months (95 % confidence interval [CI] 5.1–7.9) and 14.5 months (95 % CI 7.4–21.4), respectively. The overall response rate was 57.8 %. A CR was achieved in two patients (4.4 %) and a PR in twenty-four (53.3 %). The baseline characteristics of the study population, including the response to first-line GP chemotherapy, are presented in Table 1.

**Table 1:** Patient characteristics

Sex	Patients (%)
Female	0 (0.0 %)
Male	45 (100 %)
Age, median (year, range)	65 (60–72)
Primary tumor site	
Bladder	30 (66.7 %)
Ureter or renal pelvic	6 (13.3 %)
Ureter or renal pelvic with bladder	9 (20.0 %)
Nodal status	
N0	6 (13.3 %)
N1	8 (17.8 %)
N2	19 (42.2 %)
N3	12 (26.7 %)
Distant metastatic site at the time of MVAC administration	
Lung	21 (46.7 %)
Bone	16 (35.6 %)
Liver	9 (20.0 %)
Other	4 (8.9 %)
Absence	16 (35.6 %)
Single	13 (28.9 %)
Multiple	16 (35.6 %)
ECOG performance status (PS)	
PS 0	20 (44.4 %)
PS 1	23 (51.1 %)
PS 2	2 (4.4 %)

*MVAC* methotrexate vinblastine Adriamycin cisplatin, *ECOG* Easton Cooperative Oncologic Group

### Characteristics of good and poor responses to previous GC chemotherapy

**Fig. 1:**
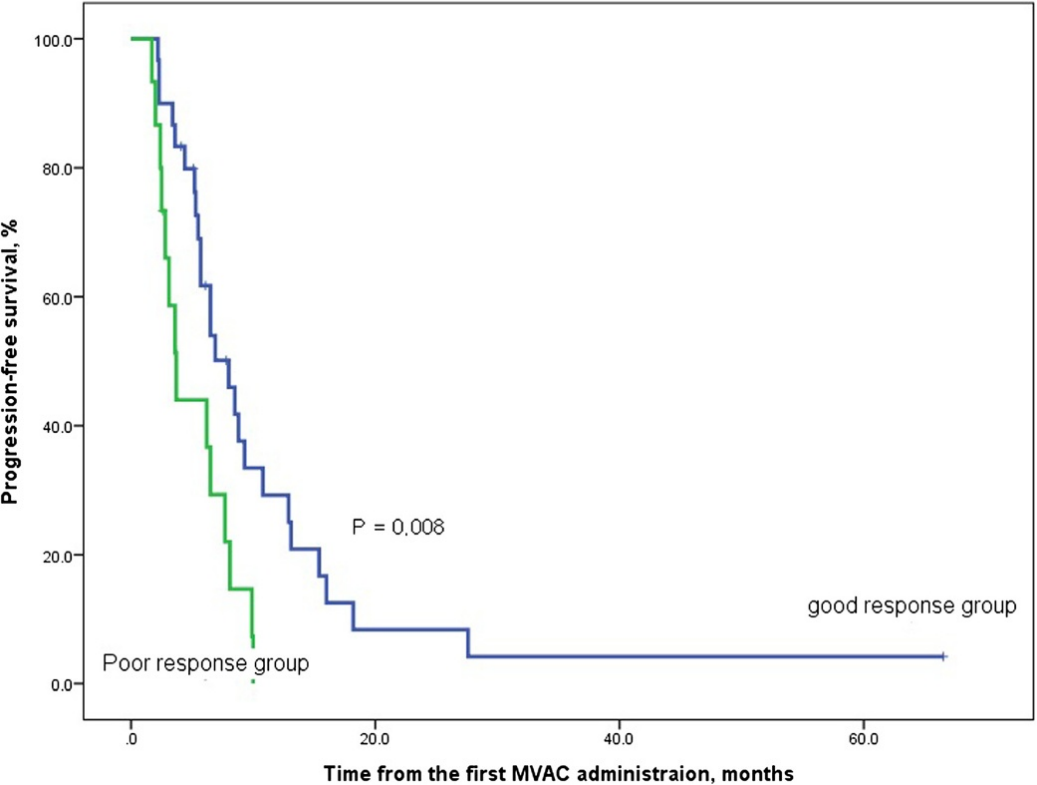
Kaplan-Meier curve for progression-free survival (%) in the good response and poor response groups. The good response group included patients who achieved a complete or partial response to first-line GP chemotherapy, whereas the poor response group included individuals with stable or progressive disease after first-line GP chemotherapy

All included patients were divided into two groups: a good response group and a poor response group, according to the response to first-line GP chemotherapy. The characteristics of the two groups are presented in Table 2. The median treatment-free intervals (TFI) between GP and MVAC chemotherapies for the good and poor response groups were 2.5 and 1.7 months, respectively. There were significant differences in haemoglobin and serum bilirubin levels between the two groups, but not in the variable related to the response to first-line GP chemotherapy (TTP and the number of cycles of first-line GP chemotherapy). The PFS of patients in each of the two groups was calculated using the Kaplan-Meier method. The median PFS after second-line MVAC chemotherapy was 8.0 months (95 % CI 5.4–10.7) in the good response group and 3.7 months (95 % CI 2.6–4.8) in the poor response group. The results of the log rank test indicated a statistically significant difference (*p* = 0.008) in PFS between the two groups (Fig. 1).

**Table 2:** Comparison of several factors for patients who were divided by the response to first-line GP chemotherapy

	Good (*n* = 30)	Poor (*n* = 15)	*P*
Age, median (year, range)	64.0 (58.5–73.0)	66.0 (64.0–71.0)	0.289
Type of platinum compound used as the first-line			0.695
Cisplatin	23 (76.7 %)	13 (86.7 %)	
Carboplatin	7 (23.3 %)	2 (13.3 %)	
Response to the first-line GP chemotherapy			-
CR	11 (36.7 %)	-	
PR	19 (63.3 %)	-	
SD	-	5 (33.3 %)	
PD	-	10 (66.6 %)	
TTP of the first-line GP chemotherapy, median (months, range)	11.6 (7.8–14.2)	3.13 (2.2–5.0)	**<0.001**
Cycles of the first-line GP chemotherapy, median (range)	6.0 (6.0–7.3)	4.0 (3.0–4.0)	**<0.001**
TFI between GP and MVAC, median (months, range)	2.5 (0.7–9.9)	1.7 (0.6–3.4)	0.202
Primary tumor site			>0.999
Bladder	20 (66.7 %)	10 (66.7 %)	
Ureter or renal pelvic	4 (13.3 %)	2 (13.3 %)	
Ureter or renal pelvic with bladder	6 (20.0 %)	3 (20.0 %)	
Clinical N stage			0.453
N0	4 (13.3 %)	2 (13.3 %)	
N1	5 (16.7 %)	3 (20.0 %)	
N2	11 (36.7 %)	8 (53.3 %)	
N3	10 (33.3 %)	2 (13.3 %)	
Distant metastatic site at the time of MVAC administration			
Lung	15 (50.0 %)	6 (40.0 %)	0.752
Bone	10 (33.3 %)	6 (40.0 %)	0.746
Liver	8 (26.7 %)	1 (6.7 %)	0.234
Others	4 (13.3 %)	- (0.0 %)	0.285
Hemoglobin (g/dL, range)	11.3 (10.5–11.9)	11.0 (9.8–11.3)	**0.032**
Absolute neutrophil count (/dL, range)	3751.5 (2733.0–5337.0)	3524.0 (2415.0–5252.0)	0.596
Platelet count, 10^3^(/dL, range)	219.5 (184.8–291.5)	216.0 (169.0–311.0)	0.952
eGFR (ml/min, range)	62.6 (52.6–75.3)	71.5 (47.0–84.7)	0.739
Serum bilirubin (mg/dL, range)	0.4 (0.3–0.6)	0.3 (0.3–0.4)	**0.047**
Serum albumin (g/dL, range)	4.0 (3.8–4.4)	3.8 (3.5–4.2)	0.159
ECOG performance status (PS)			0.275
PS 0	12 (40.0 %)	8 (53.3 %)	
PS 1	16 (53.3 %)	7 (46.7 %)	
PS 2	2 (6.7 %)	- (0.0 %)	

*GP* gemcitabine platinum, *CR* complete response, *PR* partial response, *SD* stable disease, *PD* progression disease, *TTP* time to progression, *TFI* treatment free interval, *MVAC* methotrexate vinblastine Adriamycin cisplatin, *eGFR* estimated glomerular filtration rate, *ECOG* Easton Cooperative Oncologic Group, Boldface significant 2-tailed

### Prognostic predictors of PFS after MVAC chemotherapy

Univariate and multivariate Cox’s regression analyses were performed to identify the independent predictive factors of PFS after MVAC chemotherapy. The results of the univariate and multivariate analyses demonstrated that age (hazard ratio [HR] = 1.047, *p* = 0.033) and response to first-line GC chemotherapy (HR = 2.500, *p* = 0.012) were predictors of PFS after MVAC chemotherapy (Table 3).

**Table 3:** Predictors of progression-free survival

Variables	HR	95 % CI	*P*
Univariate analysis			
Age	1.046	1.004–1.089	**0.031**
Type of platinum compound used as the first-line (cisplatin vs carboplatin)	1.670	0.750–3.718	0.209
TTP of the first-line GP chemotherapy	0.951	0.885–1.022	0.175
Cycles of the first-line GP chemotherapy	0.947	0.834–1.076	0.404
TFI between GP and MVAC	0.991	0.943–1.042	0.735
Primary tumor site			
Bladder vs ureter or renal pelvis/ ureter or renal pelvis with bladder	1.525	0.782–2.975	0.216
Bladder/ ureter or renal pelvis vs ureter or renal pelvis with bladder	1.735	0.806–3.733	0.159
Nodal status			
N (0 vs 1,2,3)	1.074	0.413–2.789	1.074
N (0,1 vs 2,3)	0.712	0.354–1.431	0.340
N (0,1,2 vs 3)	0.541	0.246–1.193	0.128
Metastatic site			
M0 vs M1	1.335	0.670–2.658	0.411
Lung	1.502	0.798–2.872	0.207
Bone	1.207	0.631–2.310	0.569
Liver	1.854	0.862–3.987	0.114
Others	1.056	0.371–3.007	0.919
Multi-organ metastasis (absence vs presence)	1.696	0.874–3.294	0.118
Hemoglobin	0.846	0.661–1.082	0.182
Absolute neutrophil count	1.000	1.000–1.000	0.823
Platelet count	1.001	0.997–1.004	1.001
eGFR	1.000	0.979–1.021	0.989
Serum bilirubin	0.529	0.077–3.630	0.517
Serum albumin	0.417	0.161–1.078	0.071
ECOG performance status (0 vs 1,2)	1.333	0.699–2.543	0.382
Response to first-line GP	2.520	1.245–5.015	**0.010**
Multivariate			
Age	1.047	1.004–1.093	**0.033**
Response to first-line GP	2.500	1.228–5.098	**0.012**

*HR* hazard ratio*, CI* confidence interval*, TTP* time to progression, *GP* gemcitabine platinum, *TFI* treatment free interval, *MVAC* methotrexate vinblastine Adriamycin cisplatin, *eGFR* estimated glomerular filtration rate, *ECOG* Easton Cooperative Oncologic Group, Boldface significant 2-tailed

### Toxicity

Anaemia, neutropenia, and thrombocytopenia ≥ grade 3 developed in 25, 29, and 20 patients, respectively. A blood transfusion or granulocyte colony-stimulating factor was administered to patients who experienced severe haematological complications. Alopecia was the most common non-haematological toxicity. Most of the non-haematological toxicities were not life threatening and were tolerated by the patients. The toxicities experienced by the patients are presented in Table 4.

**Table 4:** Toxicities

Toxicity	All grade (%)	Grade ≥3 (%)
Hematologic		
Anemia	34 (66.7 %)	25 (49.0 %)
Neutropenia	40 (78.4 %)	29 (56.9 %)
Thrombocytopenia	31 (60.8 %)	20 (39.2 %)
Non-hematologic		
Mucositis	7 (13.7 %)	4 (7.8 %)
Alopecia	44 (86.3 %)	-
Nausea/vomiting	35 (68.6 %)	3 (5.9 %)
Anorexia	28 (56.9 %)	2 (3.9 %)
Diarrhea/constipation	5 (9.8 %)	-
Fatigue	10 (19.6 %)	-
Infection	10 (19.6 %)	8 (15.7 %)

## Discussion

MVAC elicited a significant response rate in GP-failed patients with advanced urothelial cancer in the current study, which is consistent with the results reported in previous studies using MVAC in the second-line setting. Since Han et al. reported the efficacy and toxicity of MVAC as a second-line chemotherapy, [13] several additional studies have assessed the efficacy of this second-line treatment regimen [10–12]. The current study demonstrated that MVAC resulted in a significantly higher response rate and longer PFS after the failure of GP in patients with advanced UC who responded to GP as the first-line chemotherapy compared with non-responders. The response rate and median PFS of patients administered second-line MVAC after GP was 77 % and 8.0 months in responders compared with 40 % and 3.7 months in non-responders, respectively. This suggests that re-challenge using a platinum-based regimen could induce a high response rate and durable PFS after the initial failure of first-line platinum-based chemotherapy in patients with advanced UC. In addition, a higher response rate and longer PFS could be achieved by patient selection.

Several studies have reported efforts to find an effective chemotherapeutic regimen after the failure of platinum-based chemotherapy for patients with metastatic, recurrent and inoperable UC [16]. Among the non-platinum-based combination regimens, paclitaxel and gemcitabine have achieved the greatest response rate and PFS of the regimens tested to date in a phase 2 study [17]. Paclitaxel and gemcitabine chemotherapy yielded a 60 % response rate and a 14.4-month median OS. However, 25 of the 41 patients had been treated using first-line platinum-based chemotherapy in a neo-adjuvant or adjuvant setting, and the response rate was only 27 % in the metastatic setting. Bellmunt et al., reported that Vinflunine improved median PFS and OS in a phase 3 study [18]. However, Vinflunine yield only and 8.6 % response rate, and did not demonstrate fully satisfactory survival benefits. Consequently, no satisfactory standard therapy has been found in a second-line setting [19].

Several investigators have questioned whether failure of one platinum-based chemotherapy means true resistance to another platinum-based chemotherapy in UC patients, because different combinations of chemotherapeutic agents exert different synergistic effects on cancer cells, even if they are all based on platinum [10–13, 20]. Edeline et al. and Karadimou et al. suggested that patients with a history of first-line GP chemotherapy in an adjuvant setting and a relatively better ECOG performance status had a more favourable response after second-line MVAC chemotherapy [11, 12]. In addition, Han et al. suggested that patients with a complete or partial response to first-line platinum-based chemotherapy were more likely to show a favourable response to a second-line platinum-based chemotherapy [13]. Gondo et al. also supported a relationship between the responses to first-line and second-line platinum-based chemotherapy in their study on the efficacy and safety of GC as second-line chemotherapy after the failure of first-line MVAC [20]. However, all these studies were limited by a small sample size. The current study is the first to report that the response to first-line GP is a statistically significant predictive marker for response rate and PFS after second-line MVAC, and it also demonstrates that proper patient selection could achieve a higher response rate and longer PFS in patients with advanced UC.

There are several possible explanations for the re-response phenomenon to platinum-based chemotherapy in patients who failed a first platinum-based chemotherapy. Even after UC cells acquire platinum resistance and the disease progresses, the cells might acquire total resistance to gemcitabine but have incomplete resistance to platinum. Several authors suggested that the synergic effects of combination chemotherapy appear to be able to overcome the resistance of first-line platinum-based chemotherapy in ovarian and lung cancer [21–23]. Combination therapy using more than two agents induces synergistic anti-cancer effects, and different combinations of chemotherapeutic agents can induce different synergies. The MVAC regimen might induce different synergistic effects from those of the GP regimen in UC. Methotrexate, vinblastine and Adriamycin might support the anti-cancer activity of cisplatin differently from that of gemcitabine; therefore, these different effects can affect the response of GP-failed patients to MVAC.

One of the problems with second-line treatment of advanced UC patients is that performance generally deteriorates after the failure of first-line chemotherapy due to cancer progression, delayed recovery of marrow function or decreased organ function. This limits the choice of chemotherapeutic agents in the second-line setting, because the lack of response to second-line treatment can be fatal if it results in further progression of metastases and deterioration of general performance and organ function. Therefore, the appropriate selection of a second-line therapeutic regimen is critical in patients with advanced UC. Additionally, patients receiving MVAC chemotherapy could experience a variety of complications. Several studies have reported MVAC chemotherapy-based toxicities [24–28]. Haematological toxicities developed in ~70 % of the patients in the current study, and many different non-haematological toxicities also occurred. Because these complications can be life threatening, the use of second-line MVAC chemotherapy should be considered carefully and used only in patients who can benefit from this approach. Therefore, accurately predicting patients who will have a favourable response to second-line MVAC chemotherapy is critical.

The main strength of the current study was the identification of independent predictors of PFS in patients who received second-line MVAC chemotherapy, even though the results might have been affected by the retrospective nature of the study and the small number of patients included in the analysis. These results need to be confirmed and validated by analysing data from a larger prospective study.

## Conclusions

The response to first-line GP is an important predictive and prognostic factor for second-line MVAC in patients with advanced UC. MVAC chemotherapy should be considered as second-line treatment for advanced UC patients who originally responded to GP.

## Abbreviations

UC: Urothelial carcinoma
GP: Gemcitabine and platinum
GC: Gemcitabine and cisplatin
MVAC: Methotrexate, vinblastine, Adriamycin and cisplatin
ECOG PS: Eastern Cooperative Oncology Group performance status
eGFR: Estimated glomerular filtration rate
PFS: Progression-free survival
OS: Overall survival
TTP: Time to progression
TFI: Treatment-free interval
CR: Complete response
PR: Partial response
SD: Stable disease
PD: Progression disease
